# Incidence of hematologic malignancies and mortality associated with GLP-1 receptor agonist and SGLT2 inhibitor use in type 2 diabetes mellitus: results of a retrospective cohort study of electronic health records

**DOI:** 10.1016/j.eclinm.2025.103749

**Published:** 2026-01-14

**Authors:** Eric Edward Irons, Keri Ann Pfeil, Jaime Abraham Perez, Koen van Besien

**Affiliations:** a Division of Hematology, Department of Medicine, University Hospitals Cleveland Medical Center, Cleveland, USA; b University Hospitals Center for Clinical Research, University Hospitals Cleveland Medical Center, Cleveland, USA

**Keywords:** Glucagon-like peptide 1 receptor agonist, Sodium glucose cotransporter 2 inhibitor, Obesity, Diabetes mellitus, Multiple myeloma

## Abstract

**Background:**

Individuals with type 2 diabetes mellitus are treated with a growing variety of medications targeting multiple metabolic pathways. In recent years, incretins such as the GLP-1 receptor agonists have proven effective in the control of hyperglycemia but also in weight loss, where they are now separately approved as a treatment for obesity. Given the importance of obesity and metabolic syndrome as risk factors for malignancy, the impact of GLP-1 receptor agonists on cancer risk is of increasing interest. Here, we performed an analysis of the impact of GLP-1 receptor agonists and SGLT2 inhibitors on cancer risk and mortality.

**Methods:**

We performed a multicenter retrospective cohort study using the TriNetX database of electronic health records between 2019 and 2024, including patients with a diagnosis of type 2 diabetes mellitus. Individuals prescribed GLP-1 receptor agonists, SGLT2 inhibitors, or neither were compared for the incidence of four obesity-related hematologic malignancies using a Cox proportional hazards model. Similar analysis was applied to mortality associated with these medication classes in individuals with type 2 diabetes mellitus and the hematologic malignancies under study.

**Findings:**

GLP-1 receptor agonist use is associated with a significantly decreased risk of multiple myeloma (HR 0.64, p = 0.01), but not chronic myeloid leukemia (HR 1.06, p = 0.857), acute myeloid leukemia (HR 0.81, p = 0.354), or myelodysplastic syndrome (HR 0.98, p = 0.996). This effect was preserved in subgroups with BMI >30 and HbA1c >8%. Further, we find that in patients with multiple myeloma and acute myeloid leukemia, SGLT2 inhibitor use is associated with a significantly increased risk of mortality, independent of heart or kidney failure (MM HR 2.27, p < 0.001, AML HR 2.00, p = 0.006).

**Interpretation:**

Our results strengthen the association between metabolic disease and multiple myeloma yet call for prospective investigation into the use of SGTL2 inhibitors in patients with specific hematologic neoplasms.

**Funding:**

10.13039/100000002National Institutes of Health.

**Research in context**

**Evidence before this study**

Obesity has been associated with an increased risk of certain hematologic cancers. Although the weight loss-inducing GLP-1 receptor agonists are associated with risk reduction for obesity-related cancers, it is unclear whether they also alter the risk of hematologic malignancies. Prior to this study, we searched Pubmed for “hematologic cancer” and “GLP-1 receptor agonist” and found 187 results between 2015 and 2025. High-quality evidence suggested a reduced risk of progression from monoclonal gammopathy of undetermined significance to multiple myeloma among US veterans with diabetes on GLP-1 RA therapy, and a lower incidence of multiple myeloma among GLP-1 RA users compared to insulin users.

**Added value of this study**

We show that, among four hematologic malignancies, GLP-1 RA use was associated with reduced risk of multiple myeloma, including in those with obesity and poorly controlled diabetes. However, GLP-1 RA use did not modify risk of other obesity-related hematologic malignancies. SGLT2 inhibitor use was also associated with increased risk for mortality in those with type 2 diabetes mellitus and either multiple myeloma or acute myeloid leukemia, independent of congestive heart failure.

**Implications of all the available evidence**

GLP-1 RA therapy is a reasonable front-line choice for type 2 diabetes mellitus treatment in those with elevated risk of multiple myeloma, including older adults, certain ethnic or racial groups, or those with MGUS. GLP-1 RA use also appears to be safe in those with a diagnosis of AML, CML, MM, and MDS. However, SGLT2 inhibitors should be used with caution in those with AML and MM, though further studies are needed to clarify why they are associated with higher mortality.

## Introduction

The clinical indications for Glucagon-like peptide 1 receptor agonists (GLP-1 receptor agonists) include type 2 diabetes and obesity. Though first approved in 2005 for use in type 2 diabetes, analysis of data from the LEAD trials showed that they also induced significant weight loss.^1^, ^2^, ^3^ Further positive results from the SCALE trial in pre-diabetics and obese non-diabetics led to the 2014 approval of liraglutide in non-diabetics with obesity.^4^ Today, the benefits of GLP-1 receptor agonists also encompass mitigation of risk for Major adverse cardiovascular events (MACE), myocardial infarction, stroke, cardiovascular death, peripheral artery disease, and heart failure.^5^

Obesity is a risk factor for malignancy, with some 11.9% of male and 13.1% of female cancers attributed to obesity worldwide. The risk of cancer at thirteen sites, including endometrium, esophagus, kidney, pancreas, liver, stomach, meninges, multiple myeloma, colon/rectum, breast, ovary, gallbladder, and thyroid, has been associated with obesity.^6^ This has sparked interest in the potential impact of weight-loss medications on obesity-related cancer risk. Based on post-approval analysis of reported adverse events from the Federal Adverse Event Event (FAERS) database, GLP-1 receptor agonists have proven generally to be safe in regards to risk for malignancy, with no evidence of drug-attributable increase in risk for overall malignancy (proportional reporting ratio 0.83), but raised risk of specific malignancies, including medullary thyroid cancer (PRR 27.43), papillary thyroid cancer (PRR 8.68), pancreatic neoplasms (PRR 9.86), and islet cell neoplasms (PRR 2.86), leading to a black box warning for individuals with either Multiple endocrine neoplasia (MEN) 2 syndrome or a family history of medullary thyroid cancer.^7^

However, GLP-1 receptor agonist use has also been associated with reduced risk of developing 10 of 13 obesity-associated cancers (gallbladder cancer, meningioma, pancreatic cancer, hepatocellular cancer, ovarian cancer, colorectal cancer, multiple myeloma, esophageal cancer, endometrial cancer, and kidney cancer) in a large multi-institutional analysis of electronic health records, when compared to insulin.^8^ Further analysis has shown that GLP-1 receptor agonist use is in fact associated with consistently reduced risk of pancreatic cancer compared to use of 6 other classes of antidiabetic medications, and the benefits were greater in those with concurrent obesity and tobacco use.^9^ A similar analysis for colorectal cancer showed that GLP-1 receptor agonist use was associated with consistently reduced risk of cancer when compared to seven non-GLP-1 receptor agonist classes of diabetes medications.^10^

Among the hematologic malignancies, a Veterans Affairs Health System analysis of 1097 individuals with diabetes and monoclonal gammopathy of undetermined significance (MGUS) found that those who had been exposed to GLP-1 receptor agonist medications had a reduced risk (HR = 0.45) of progression to multiple myeloma.^11^ This is consistent with another analysis in which use of metformin, another diabetes medication capable of inducing weight loss, was associated with a reduced risk of MGUS progression to multiple myeloma (HR = 0.47).^12^ Another study has reported a decreased incidence of a variety of hematologic malignancies in type 2 diabetics using GLP-1 receptor agonists, including myeloid and lymphoid leukemias, non-Hodgkin's lymphoma, myelodysplastic syndromes, myeloproliferative neoplasms, monoclonal gammopathy, and multiple myeloma.^13^ The influence of GLP-1 receptor agonists on pathogenesis of hematologic malignancies and any potential impact they have on survival remains to be fully understood.

In diabetic patients who develop cancer, there is little consensus regarding whether preexisting treatment plans for diabetes should be modified to protect against new risk factors associated with malignancy. Weight-loss diabetes medications such as metformin and GLP-1 receptor agonists may exacerbate cancer-associated cachexia and frailty, whereas thiazolidinediones may compound chemotherapy-associated risks for cardiomyopathy and congestive heart failure. Sodium-glucose Cotransporter 2 inhibitor medications (SGLT2 inhibitors), which promote glucosuria, are known to raise the risk of urinary tract infection and candidiasis, risks that may be unacceptable in an immunocompromised population. A small retrospective study of diabetics with multiple myeloma at a single center found that there was a 42.4% rate of SGLT2 inhibitor discontinuation, with 21.4% attributed to worsening renal function, but no evidence of increased risk of infection.^14^ It has been suggested that for cancer patients undergoing anthracycline-based chemotherapy, SGLT2 inhibitor use is associated with a lower incidence of subsequent cardiac events and lower overall mortality.^15^ Further work will be necessary to define the balance between risk and benefit here.

Here, we present de-identified electronic health record data from the TriNetX Research Database analyzing the risks associated with GLP-1 receptor agonist and SGLT2 inhibitor medication use among individuals with type 2 diabetes mellitus. Focusing on four common hematologic malignancies (acute myeloid leukemia, multiple myeloma, chronic myeloid leukemia, and myelodysplastic syndrome), we report that GLP-1 receptor agonist use was associated with a reduced risk of multiple myeloma, whereas neither drug class was associated with significantly altered risk for hematologic malignancy in other comparisons. The positive impact of GLP-1 receptor agonists persisted in subgroups of poorly controlled and obese patients. Further, in individuals diagnosed with both type 2 diabetes mellitus and either acute myeloid leukemia, chronic myeloid leukemia, or multiple myeloma, SGLT2 inhibitor use was associated with an increased risk of mortality. Among those with multiple myeloma, SGLT2 inhibitor use increased mortality risk in both individuals with and without congestive heart failure.

## Methods

### Patient Selection

We performed a retrospective cohort study using a de-identified national database with 91 contributing health care organizations (HCO), including over 129 million patients (TriNetX Research Network, Cambridge, MA; date of data access: September 6, 2024).

### Ethics

This retrospective study was exempt from informed consent and IRB review. The data reviewed was a secondary analysis of existing data, did not involve intervention or interaction with human subjects, and was de-identified per the de-identification standard defined in Section §164.514(a) of the HIPAA Privacy Rule.

We identified all individuals with at least two coded diagnoses of type 2 diabetes (ICD-10: E11) between September 6, 2019 and September 6, 2024 with available Hemoglobin A1c (HbA1c), body mass index (BMI), demographic, and covariate data (as used in the weighting described below). We categorized patients into cohorts based on receiving GLP-1 receptor agonist only, SGLT-2 inhibitor only or neither medication, where diabetes without either medication was used as the reference. Analyses focused on the association between these medications and the outcomes of Acute myeloid leukemia, Multiple myeloma, Chronic myeloid leukemia, Myelodysplastic syndrome and mortality.

### Statistical analysis

A time-dependent Cox proportional hazard model was used to determine the risk of Acute myeloid leukemia, Multiple myeloma, Chronic myeloid leukemia, and Myelodysplastic syndrome after type 2 diabetes diagnosis. In all cases, the proportional hazards assumption was verified prior to analysis. Models included GLP-1 receptor agonist and SGLT-2 inhibitor medications as a time-varying covariate and race and ethnicity as fixed covariates. Additional analyses included a cohort with HbA1c >8% and another cohort with BMI >30. Patients were followed from type 2 diabetes diagnosis to hematological malignancy or last known encounter, whichever occurred first. Event time was defined as first coded diagnosis of the malignancies under study.

We also investigated the association between GLP-1 receptor agonist or SGLT2 inhibitor use and risk for mortality in these four hematologic neoplasms. Here, cohorts included individuals with type 2 diabetes mellitus, who had started either GLP-1 receptor agonist or SGLT2 inhibitor therapy and were diagnosed with one of the four hematologic malignancies under study. For these analyses, the reference used was those with diabetes and one of the malignancies and not on either class of medications. A Cox proportional hazard model was used to investigate survival after diagnosis of malignancy. Patients were followed from diagnosis of malignancy to death or until their last known encounter, which ever occurred first. Event time was defined as death. Observations were censored at 2 years for Acute myeloid leukemia, 5 years for Multiple myeloma, 10 years for Chronic myeloid leukemia, and 3 years for Myelodysplastic syndrome. Covariates in the models included race, ethnicity, and heart failure. In models where heart failure was significant, the analysis was rerun for those without heart failure and those with heart failure to see if the relationship between the outcome of death and medication was dependent on having heart failure. Estimates of hazard ratios (HR), and 95% confidence intervals (CI) were used to describe the hazard of the outcome for all analyses. To balance for important potential confounders of the relationship between medication use and study outcomes, all models used inverse probability of treatment weighting (IPTW) based on age, sex, nicotine dependance, history of cerebral infarction, history of chronic ischemic heart disease, chronic kidney disease (CKD), and hypertension. The weighted sample was balanced (absolute standardized mean differences <0.1) on all variables included in the weighted models. p-values of α < 0.05 were considered statistically significant.

All analyses were completed using R version 4.3.3 (R Core Team (2024). _R: A Language and Environment for Statistical Computing. R Foundation for Statistical Computing, Vienna, Austria. https://www.R-project.org/.)

### Role of funding source

This project was supported by the Clinical and Translational Science Collaborative of Northern Ohio which is funded by the National Institutes of Health, National Center for Advancing Translational Sciences, Clinical and Translational Science Award grant, UM1TR004528. The content is solely the responsibility of the authors and does not necessarily represent the official views of the NIH.

## Results

**Fig. 1:**
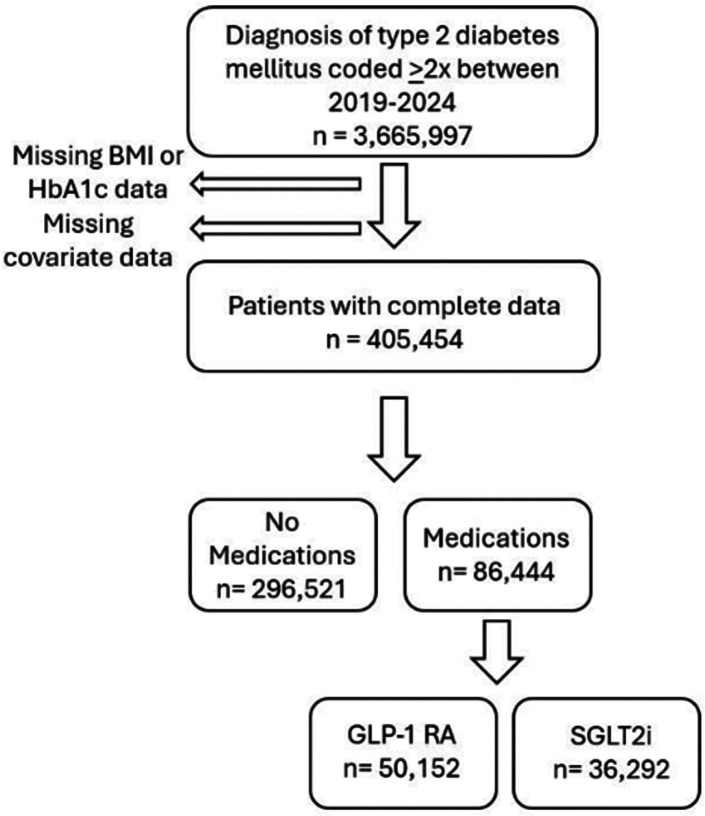
Sample size flow chart. Among the full population of patients with at least 2 coded diagnoses of type 2 diabetes mellitus in the period of 2019–2024, those missing concurrent body mass index (BMI) or hemoglobin A1c (HbA1c) data were excluded. Individuals without data on covariates, including age, sex, race, ethnicity, heart failure, nicotine dependence, hypertension, chronic kidney disease, chronic ischemic heart disease, and cerebral infarction were also excluded. The final cohort for analysis consisted of 405,454 individuals, of which 296,521 did not use either medication type, 50,152 used GLP-1 RAs, and 36,292 used SGLT2 inhibitors.

To understand how the use of GLP-1 receptor agonists impacts the risk of hematologic neoplasms, we utilized the TriNetX database, which contains coded diagnostic, treatment, and laboratory data for 129 million individuals. The TriNetX database has been extensively utilized by groups around the world for large-scale epidemiologic studies, and the methods we employed in this study represent the standard in generating representative results of highest possible accuracy, as demonstrated in recent publications.^16^^,^^17^ Since GLP-1 receptor agonists have only recently been used in obesity, we limited our analysis to patients with at least two coded diagnoses of type 2 diabetes between 2019 and 2024 (3,665,997 individuals). After exclusion of individuals without available body mass index (BMI), hemoglobin A1c (HbA1c), or covariate data, we analyzed raw data from a total of 405,454 individuals, including 50,152 prescribed a GLP-1 receptor agonist (namely dulaglutide, exenatide, semaglutide, liraglutide, or tirzepatide). We expected that GLP-1 receptor agonist use roughly selects for those with moderate to severe disease, with an increased proportion of obese individuals, given the benefits of this drug class for weight loss. As an approximate biological negative control, we included a parallel analysis of 36,292 type 2 diabetes patients using SGLT2 inhibitors (bexagliflozin, canagliflozian, empagliflozin, dapagliflozin, and ertugliflozin), which are also first-line oral agents for diabetes, but act by a different, insulin-independent mechanism, without significant effects on tissue adiposity. In both cases, use of GLP-1 receptor agonist or SGLT2 inhibitor medications must have been preceded by a diagnosis of type 2 diabetes. Both of these groups were then compared to 296,521 individuals with type 2 diabetes that were not prescribed drugs from either class. A sample size flow chart is included in Fig. 1.

Baseline patient characteristics are shown in Tables 1 and 2. Compared to non-users, GLP-1 receptor agonist users were younger (51.9 ± 13.8 vs 57.8 ± 16.5), more often female (60.1% vs 50.9%), had higher average BMI (36.2 ± 6.8 vs 31.4 ± 7.2), less chronic ischemic heart disease (7.7% vs 13.4%), less chronic kidney disease (8.4% vs 12.2%), and less heart failure (3.5% vs 7.1%). Compared to the non-users, SGLT2 inhibitor users were less often female (39.3% vs 50.9%), had more chronic ischemic heart disease (16.2% vs 13.4%) and more heart failure (11.0% vs 7.1%). Both GLP-1 receptor agonist and SGLT2 inhibitor cohorts had higher average HbA1c values (GLP-1 7.4 ± 2.2, SGLT2 7.6 ± 2.3, non-users 6.9 ± 2.0) and were less likely to have a history of cerebral infarction (GLP-1 3.5%, SGLT2 4.3%, non-users 6.3%).

**Table 1:** Baseline characteristics of subjects with diabetes mellitus who use GLP-1 receptor agonists or neither GLP-1 receptor agonists and SGLT2 inhibitors.

Characteristic	Overall N = 346,673^a^	No medication use N = 296,521^a^	GLP-1 receptor agonist use N = 50,152^a^	p-value^b^
**Age**	56.9 ± 16.3	57.8 ± 16.5	51.9 ± 13.8	**<0.001**
**Sex**				**<0.001**
Male	165,668 (47.8%)	145,665 (49.1%)	20,003 (39.9%)	
Female	181,005 (52.2%)	150,856 (50.9%)	30,149 (60.1%)	
**Race**				**<0.001**
White	239,417 (69.1%)	204,885 (69.1%)	34,532 (68.9%)	
Black or African American	66,834 (19.3%)	56,651 (19.1%)	10,183 (20.3%)	
Other race	40,422 (11.7%)	34,985 (11.8%)	5437 (10.8%)	
**Ethnicity**				**<0.001**
Not Hispanic or Latino	308,803 (89.1%)	263,811 (89.0%)	44,992 (89.7%)	
Hispanic or Latino	37,870 (10.9%)	32,710 (11.0%)	5160 (10.3%)	
**Nicotine dependance**	116,041 (33.5%)	101,629 (34.3%)	14,412 (28.7%)	**<0.001**
**BMI**	32.1 ± 7.3	31.4 ± 7.2	36.2 ± 6.8	**<0.001**
**A1c**	7.0 ± 2.1	6.9 ± 2.0	7.4 ± 2.2	**<0.001**
**Cerebral infarction**	20,425 (5.9%)	18,684 (6.3%)	1741 (3.5%)	**<0.001**
**Chronic ischemic heart disease**	43,610 (12.6%)	39,728 (13.4%)	3882 (7.7%)	**<0.001**
**Chronic kidney disease (CKD)**	40,492 (11.7%)	36,277 (12.2%)	4215 (8.4%)	**<0.001**
**Essential (primary) hypertension**	162,005 (46.7%)	139,111 (46.9%)	22,894 (45.6%)	**<0.001**
**Heart failure**	22,820 (6.6%)	21,068 (7.1%)	1752 (3.5%)	**<0.001**

p-values are shown comparing users of a particular medication class to those who do not use either.

a n (%); Mean ± SD.

b Pearson's Chi-squared test; Welch Two Sample t-test.

**Table 2:** Baseline characteristics of subjects with diabetes mellitus who use SGLT2 inhibitors or neither GLP-1 receptor agonists or SGLT2 inhibitors.

Characteristic	Overall N = 332,813^a^	No medication use N = 296,521^a^	SGLT2 inhibitor use N = 36,292^a^	p-value^b^
**Age**	58.0 ± 16.1	57.8 ± 16.5	59.6 ± 12.5	**<0.001**
**Sex**				**<0.001**
Male	167,692 (50.4%)	145,665 (49.1%)	22,027 (60.7%)	
Female	165,121 (49.6%)	150,856 (50.9%)	14,265 (39.3%)	
**Race**				**<0.001**
White	228,764 (68.7%)	204,885 (69.1%)	23,879 (65.8%)	
Black or African American	63,696 (19.1%)	56,651 (19.1%)	7045 (19.4%)	
Other Race	40,353 (12.1%)	34,985 (11.8%)	5368 (14.8%)	
**Ethnicity**				0.699
Not Hispanic or Latino	296,124 (89.0%)	263,811 (89.0%)	32,313 (89.0%)	
Hispanic or Latino	36,689 (11.0%)	32,710 (11.0%)	3979 (11.0%)	
**Nicotine dependance**	112,363 (33.8%)	101,629 (34.3%)	10,734 (29.6%)	**<0.001**
**BMI**	31.4 ± 7.1	31.4 ± 7.2	32.1 ± 6.7	**<0.001**
**A1c**	7.0 ± 2.1	6.9 ± 2.0	7.6 ± 2.3	**<0.001**
**Cerebral infarction**	20,238 (6.1%)	18,684 (6.3%)	1554 (4.3%)	**<0.001**
**Chronic ischemic heart disease**	45,598 (13.7%)	39,728 (13.4%)	5870 (16.2%)	**<0.001**
**Chronic kidney disease (CKD)**	40,009 (12.0%)	36,277 (12.2%)	3732 (10.3%)	**<0.001**
**Essential (primary) hypertension**	154,998 (46.6%)	139,111 (46.9%)	15,887 (43.8%)	**<0.001**
**Heart failure**	25,073 (7.5%)	21,068 (7.1%)	4005 (11.0%)	**<0.001**

p-values are shown comparing users of a particular medication class to those who do not use either.

p-values that were statistically significant (i.e <0.05) were bolded.

a n (%); Mean ± SD.

b Pearson's Chi-squared test; Welch Two Sample t-test.

**Fig. 2:**
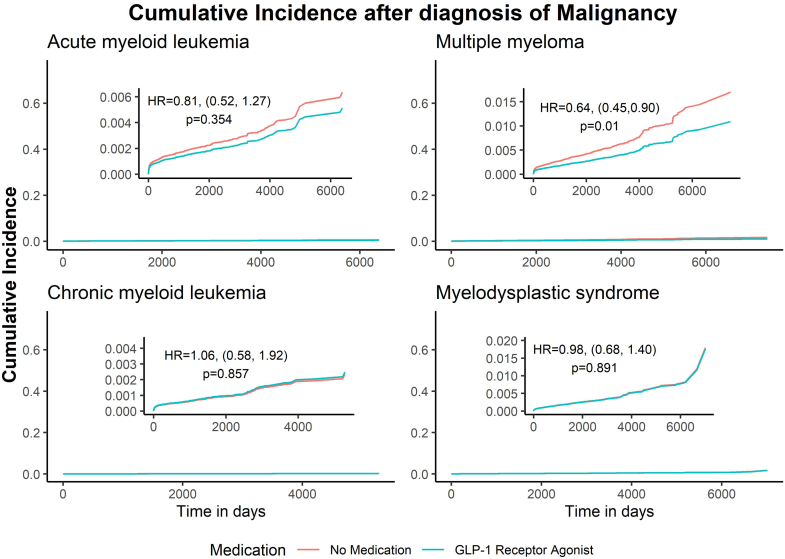
Cumulative incidence curves for diagnosis of hematologic malignancy in GLP-1 RA users. Cumulative incidence of the given hematologic malignancy from diagnosis of type 2 diabetes mellitus is presented, comparing those using GLP-1 RAs and those not using GLP-1 RA or SGLT2 inhibitors. Models were adjusted for race and ethnicity and weighted by age, sex, nicotine dependance, history of cerebral infarction, history of chronic ischemic heart disease, chronic kidney disease (CKD) and hypertension.

**Fig. 3:**
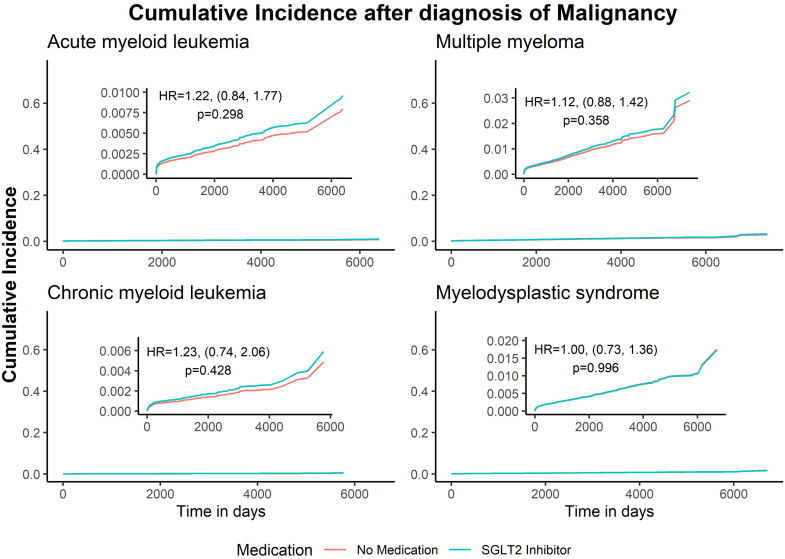
Cumulative incidence curves for diagnosis of hematologic malignancy in SGLT2 inhibitor users. Cumulative incidence of the given hematologic malignancy from diagnosis of type 2 diabetes mellitus is presented, comparing those using SGLT2 inhibitors and those not using GLP-1 RA or SGLT2 inhibitors. Models were adjusted for race and ethnicity and weighted by age, sex, nicotine dependance, history of cerebral infarction, history of chronic ischemic heart disease, chronic kidney disease (CKD) and hypertension.

Results from a time-dependent Cox proportional hazard model are presented in Table 3. We chose to focus on acute myeloid leukemia (AML), chronic myeloid leukemia (CML), myelodysplastic syndrome (MDS), and multiple myeloma (MM) as outcomes, since risk for AML, CML, and MM have been most robustly associated with obesity in previous epidemiologic studies, whereas MDS has not and served as a negative control. Our analysis shows that there was no difference in the hazard between those on either medication class compared to those that were not for most malignancies. This included AML (GLP-1 users HR 0.81, 95% CI 0.52–1.27, p = 0.354; SGLT2 inhibitor users HR 1.22, 95% CI 0.84–1.77, p = 0.298), CML (GLP-1 users HR 1.06, 95% CI 0.58–1.92, p = 0.857; SGLT2 inhibitor users HR 1.23, 95% CI 0.74–2.06, p = 0.428), and MDS (GLP-1 users HR 0.98, 95% CI 0.68–1.40, p = 0.891; SGLT2 inhibitor users HR 1.0, 95% CI 0.73–1.36, p = 0.996). However, the event rate of MM was significantly reduced in GLP-1 receptor agonist users (HR 0.64, 95% CI 0.45–0.90, p = 0.01). In contrast, there was no significant difference in multiple myeloma event rate for those on SGLT2 inhibitors (HR 1.12, 95% CI 0.88–1.42, p = 0.358). Cumulative incidence curves depicting the diagnosis of hematologic malignancy from time of diabetes mellitus diagnosis in GLP-1 RA and SGLT2 inhibitor users are shown in Figs. 2 and 3.

**Table 3:** Risk of hematologic malignancies in users of GLP-1 RAs and SGLT2 inhibitors.

	Medication	Number of patients	Event n	HR^a^	95% CI^a^	p-value
Acute myeloid leukemia	No Drug^b^^,^^c^	296,521	907	–	–	–
	GLP-1 RA	50,152	20	0.81	0.52, 1.27	0.354
	No Drug^b^^,^^d^	296,521	915	–	–	–
	SGLT2 inhibitor	36,292	32	1.22	0.84, 1.77	0.298
Multiple myeloma	No Drug^b^^,^^c^	296,521	1844	–	–	–
	GLP-1 RA	50,152	33	0.64	0.45, 0.90	**0.01**
	No Drug^b^^,^^d^	296,521	1890	–	–	–
	SGLT2 inhibitor	36,292	78	1.12	0.88, 1.42	0.358
Chronic myeloid leukemia	No Drug^b^^,^^c^	296,521	339	–	–	–
	GLP-1 RA	50,152	12	1.06	0.58, 1.92	0.857
	No Drug^b^^,^^d^	296,521	350	–	–	–
	SGLT2 inhibitor	36,292	17	1.23	0.74, 2.06	0.428
MDS	No Drug^b^^,^^c^	296,521	1209	–	–	–
	GLP-1 RA	50,152	32	0.98	0.68, 1.40	0.891
	No Drug^b^^,^^d^	296,521	1248	–	–	–
	SGLT2 inhibitor	36,292	46	1	0.73, 1.36	0.996

Hazard ratio, confidence interval and p-values from a time-dependent Cox proportional hazards model for the outcomes of hematologic neoplasms in subjects with type 2 diabetes mellitus using GLP-1 receptor agonists or SGLT2 inhibitors. Models were adjusted for race and ethnicity and weighted by age, sex, nicotine dependance, history of cerebral infarction, history of chronic ischemic heart disease, chronic kidney disease (CKD) and hypertension.

Subjects with malignancy diagnosis prior to medication were counted within the control events. This accounts for the slight difference in incidence for control GLP1- RA vs control SGLT2 inhibitor group.

p-values that were statistically significant (i.e <0.05) were bolded.

a HR, Hazard Ratio; CI, Confidence Interval.

b Patients taking neither GLP-1 RA nor SGLT2 inhibitors.

c Control group for subjects taking GLP1-RA.

d Control group for subjects taking SGLT2 inhibitors.

Since GLP-1 receptor agonist users had slightly poorer glycemic control compared to non-users, we sought to analyze the same outcomes in patients with poorly controlled diabetes (HbA1c > 8%) (Supplementary Table S1). Here, we observed that, similar to the larger cohort, the only significant association was a reduced risk of multiple myeloma in individuals using GLP-1 receptor agonists (HR 0.33, 95% CI 0.12–0.90, p = 0.03). Those using SGLT2 inhibitors did not have an altered risk of multiple myeloma (HR 1.04, 95% CI 0.62–1.72, p = 0.885). Since GLP-1 receptor agonists have widespread utility in weight loss, they are more likely to be prescribed for diabetics who are also obese, as demonstrated by a higher average BMI in GLP-1 receptor agonist users, compared to non-users. Thus, we repeated our analysis in patients with obesity (BMI >30) (Supplementary Table S2). In these analyses, we similarly saw that the only significant association was between GLP-1 receptor agonist use and a decreased hazard of multiple myeloma (HR 0.64, 95% CI 0.42–0.96, p = 0.032). Those using SGLT2 inhibitors still did not have an altered hazard of multiple myeloma (HR 1.07, 95% CI 0.77–1.47, p = 0.695). These results indicate that there is a consistent relationship between GLP-1 receptor agonist use and a decrease in hazard of multiple myeloma among individuals with type 2 diabetes mellitus, including in those with poor glucose control and those with obesity.

In order to further enhance the rigor of the analysis, we adjusted for the variables used for weighting, including race, ethnicity, age, sex, nicotine dependence, history of cerebral infarction, history of chronic ischemic heart disease, chronic kidney disease, and hypertension. The relationship between MM and GLP-1 RA use remained similar for the overall sample (HR = 0.66, CI = 0.47, 0.93, p-value = 0.018) and for those with HbA1c > 8% (HR = 0.33, CI = 0.12, 0.89, p-value = 0.028) even after adjusting for the covariates. This indicates that these findings were robust to the addition of confounders. In contrast, the subgroup analysis for those with BMI >30 for the outcome of multiple myeloma was no longer significant after including the additional covariates, the confidence interval having shifted closer to 1 (HR = 0.67, CI = 0.45,1.01, p-value = 0.056) (Supplementary Table S3).

**Fig. 4:**
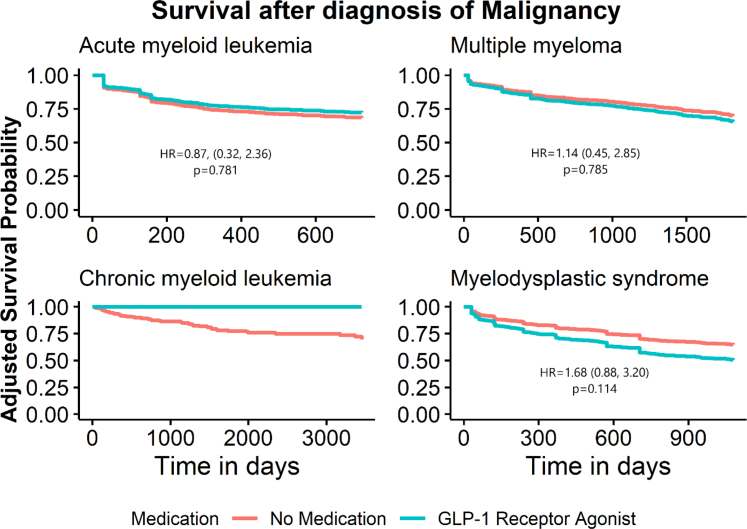
Survival after diagnosis of hematologic malignancy in individuals with type 2 diabetes taking GLP-1 RAs. Adjusted Kaplan–Meier curves for Cox proportional hazard models for those with type 2 diabetes and hematologic neoplasms with the outcome of death or last follow up, comparing those with GLP-1 RAs and without either GLP-1 RAs or SGLT2 inhibitors. Models were adjusted for race, ethnicity and heart failure and weighted by age, sex, nicotine dependance, history of cerebral infarction, history of chronic ischemic heart disease, chronic kidney disease (CKD) and hypertension.

**Fig. 5:**
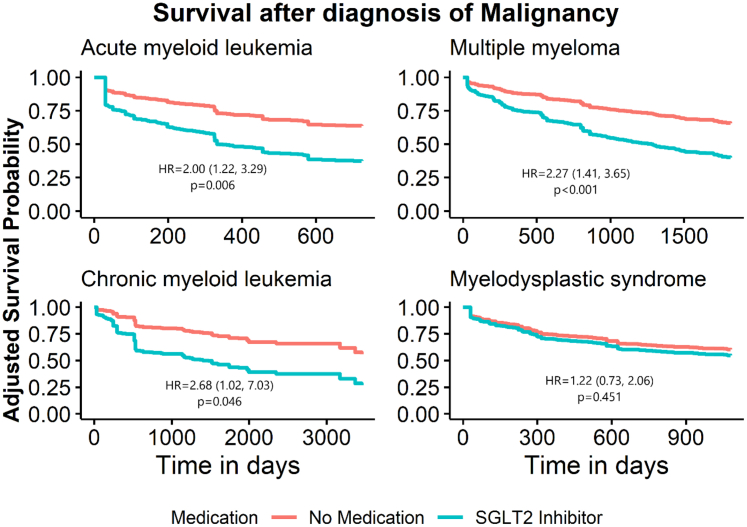
Survival after diagnosis of hematologic malignancy in individuals with type 2 diabetes taking SGLT2 inhibitors. Adjusted Kaplan–Meier curves for Cox proportional hazard models for those with type 2 diabetes and hematologic neoplasms with the outcome of death or last follow up, comparing those with GLP-1 RAs and without either GLP-1 RAs or SGLT2 inhibitors. Models were adjusted for race, ethnicity and heart failure and weighted by age, sex, nicotine dependance, history of cerebral infarction, history of chronic ischemic heart disease, chronic kidney disease (CKD) and hypertension.

We also investigated the association between GLP-1 receptor agonist or SGLT2 inhibitor use and risk for mortality in these four hematologic neoplasms. Here, we included individuals with both type 2 diabetes mellitus and at least one of the four hematologic malignancies under study, and once again weighted for age, sex, nicotine dependance, history of cerebral infarction, history of chronic ischemic heart disease, chronic kidney disease, and hypertension. Baseline characteristics are shown in Supplementary Tables S4–S11. To control for significant differences in follow-up time between cohorts, we instituted clinically relevant time limits on follow up, namely 2 years for AML, 5 years for MM, 10 years for CML, and 3 years for MDS. This was based on the expected survival of these diseases from publicly available SEER data. In our analysis, we noted significantly increased risks of mortality associated with SGLT2 inhibitors, including in AML (HR 2.00, 95% CI 1.22–3.29, p = 0.006), multiple myeloma (HR 2.27, 95% CI 1.41–3.65, p < 0.001), and CML (HR 2.68, 95% CI 1.02–7.03, p = 0.046) (Table 4). In contrast, GLP-1 receptor agonist use did not associate with altered mortality risk (Table 4). Kaplin-Meier curves depicting the risk of mortality over time in these analyses are included in Figs. 4 and 5.

**Table 4:** Risk of mortality by use of GLP-1 RA or SGLT2 inhibitors in type 2 diabetes with concurrent diagnosis of hematologic malignancy.

Malignancy	Medication	Number of patients	Event n	HR^a^	95% CI^a^	p-value
Acute myeloid leukemia	No Drug^b^^,^^c^	854	242	–	–	–
	GLP-1 RA	21	4	0.87	0.32, 2.36	0.781
	No Drug^b^^,^^d^	854	242	–	–	–
	SGLT2 inhibitor	34	16	2.00	1.22, 3.29	**0.006**
Multiple myeloma	No Drug^b^^,^^c^	1716	396	–	–	–
	GLP-1 RA	34	5	1.14	0.45, 2.85	0.785
	No Drug^b^^,^^d^	1716	396	–	–	–
	SGLT2 inhibitor	83	22	2.27	1.41, 3.65	**<0.001**
Chronic myeloid leukemia	No Drug^b^^,^^c^	307	71	–	–	–
	GLP-1 RA	13	0	–	–	–
	No Drug^b^^,^^d^	307	71	–	–	–
	SGLT2 inhibitor	16	5	2.68	1.02, 7.03	**0.046**
Myelodysplastic syndrome	No Drug^b^^,^^c^	1145	357	–	–	–
	GLP-1 RA	34	10	1.68	0.88, 3.20	0.114
	No Drug^b^^,^^d^	1145	357	–	–	–
	SGLT2 inhibitor	47	15	1.22	0.73, 2.06	0.451

Results from a Cox proportional hazards model with the outcome of mortality or last follow-up in individuals with hematologic malignancies who use GLP-1 receptor agonists or SGLT2 inhibitors. Analysis was adjusted for the covariates of race, ethnicity, and heart failure (hazard ratios not shown) and were weighted by age, sex, nicotine dependance, history of cerebral infarction, history of chronic ischemic heart disease, chronic kidney disease (CKD) and hypertension. Table presents hazard ratio, 95% confidence interval and event rate as percent of total observations. Significant p-values in bold.

a HR, Hazard Ratio; CI, Confidence Interval.

b Patients taking neither GLP-1 RA nor SGLT2 inhibitors.

c Control group for subjects taking GLP1-RA.

d Control group for subjects taking SGLT2 inhibitors.

Congestive heart failure is a strong indication for SGLT2 inhibitor use that may significantly increase the risk of mortality. The preference for SGLT2 inhibitor use in type 2 diabetes with congestive heart failure is based on extensive clinical data from the DAPA-HF, EMPEROR, DELIVER, and other trials demonstrating reduced mortality and hospitalizations in heart failure with either reduced or preserved ejection fraction (reviewed in^18^). In order to address this, we included congestive heart failure as a covariate. We found that congestive heart failure was a significant covariate associated with increased mortality among SGLT2 inhibitor users in CML (HR 7.15, 95% CI 3.26, 15.6, p = <0.001) and MM (HR 1.88, 95% CI 1.11, 3.17, p = 0.019), but not AML (HR 1.43, 95% CI 0.77, 2.66, p = 0.261) or MDS (HR 1.18, 95% CI 0.59–2.37, p = 0.64) (data not shown). To investigate this association further we created two cohorts, those without heart failure and those with heart failure, to determine if the increased mortality associated with SGLT2 inhibitor use was confounded by heart failure. Since heart failure was not a significant covariate for mortality in AML or MDS, we did not pursue further analysis of this subgroup. However, in MM, SGLT2 inhibitor use was associated with increased mortality in both those with (HR 2.62, 95% CI 1.10–6.24, p = 0.03) and without a diagnosis of congestive heart failure (HR 2.14, 95% CI 1.25–3.66, p = 0.006) (Supplementary Table S12). In CML, individuals without heart failure no longer exhibited an increased mortality associated with SGLT2 inhibitor use (HR 1.49, 95% CI 0.35–6.32, p = 0.588) (data not shown). We found that our CML analysis had the lowest fragility index of all our models (FI = 2) and given the small sample size and wide confidence intervals in this sub analysis, these results must be interpreted with caution.

Again, we adjusted for the covariates of age, sex, nicotine dependance, history of cerebral infarction, history of chronic ischemic heart disease, chronic kidney disease, and hypertension to test the robustness of the results and found that the results were preserved. For patients with AML, MM, and CML there was an increase in hazard of death for those on SGLT2 inhibitors after adjusting for the additional covariates (AML HR 2.47, CI 1.45–4.16, p < 0.001; MM HR 2.44, CI 1.51–3.95, p < 0.001; CML HR 3.46, CI 1.15–10.4, p = 0.027) (Supplementary Table S13). For those with multiple myeloma, heart failure was no longer significantly associated with an increased risk of death (HR 1.41, CI 0.74–2.72, p-value = 0.30).

In conclusion, our data argue in favor of an increased risk of death in AML and MM patients with type 2 diabetes mellitus using SGLT2 inhibitors, even when accounting for a variety of metabolic covariates, including chronic kidney disease and congestive heart failure. A directed acyclic graph representing the hypothesized causal relationships between SGLT2 inhibitors and mortality in AML, CML, and MM is provided (Supplementary Figure S1).

## Discussion

Our study adds to a rapidly growing body of literature that speaks to the efficacy of weight-loss medications in the modification of cancer risk. As might be expected, GLP-1 receptor agonist use has been most robustly associated with reduced risk of various obesity-related cancers, including colorectal and pancreatic, with recent analyses demonstrating that use is associated with variously reduced risk of 10 of 13 obesity-associated cancers.^8^, ^9^, ^10^ Here, we sought to answer the question of whether GLP-1 receptor agonist use also modifies risk of hematologic neoplasms. There is strong rationale for this hypothesis, as obesity has been repeatedly linked with various hematologic malignancies. An early large study of ∼4.5 million hospitalized veterans in the United States found that obesity was associated with higher risk of MM and AML, but not of chronic lymphocytic leukemia, CML, Hodgkin's or non-Hodgkin's lymphomas.^19^ In a large South Korean cohort study of 781,283 individuals over 10 years, obesity was weakly associated with an increased risk of non-Hodgkin's lymphoma, but not multiple myeloma or leukemia.^20^ In an aggregate study of three large twin cohorts in Sweden and Finland totaling 70,067 individuals, obesity was associated with a significantly higher risk of MM and CML, but not acute lymphoblastic leukemia.^21^ Our findings show that GLP-1 receptor agonist use is only robustly associated with a reduced risk for multiple myeloma, in agreement with a previous study of US veterans showing that GLP-1 receptor agonist use was associated with a decreased risk of progression from MGUS to multiple myeloma.^11^

Our study has several important limitations. In utilizing de-identified electronic health record data, we are unable to confirm the veracity of coded diagnoses, duration of use, filling of prescriptions, or compliance with the prescribed medications of interest. In particular, the accuracy of diagnosis in diseases of high acuity such as AML is likely impacted by access to specialized medical care, socioeconomic status, and completing appropriate diagnostic workup prior to death, which is not reflected in our data. Regarding medications, the clinical rationale for prescription of specific medications is not available to us but could conceivably confound disease incidence and mortality outcomes. Further, information about genetics, risk stratification, and treatment choices for the studied malignancies is not available to be analyzed, nor is the access to novel medications or cellular therapies likely to be equally distributed. Third, our mortality analysis does not account for malignancy-related and malignancy-independent causes of mortality, the latter of which is likely to represent a significant proportion of deaths in diseases such as CML and MM with good treatment options. Despite these limitations, the large scale of the TriNetX data allows for the best possible control over uncontrolled variability, as they are ultimately likely to be equally distributed in the analyzed cohorts.

Recently, a similar study of hematologic malignancies among GLP-1 receptor agonist users reported a significantly reduced risk of numerous neoplasms, including multiple myeloma, MGUS, myeloproliferative neoplasms, myelodysplastic syndrome, non-Hodgkins lymphoma, myeloid and lymphoid leukemias.^13^ In that study, diabetics using GLP-1 receptor agonists were compared to those using insulin or metformin, which raises important considerations. Insulin use selects for individuals with poor glycemic control and thus may introduce significant variation in severity of diabetes. Further, insulin is adipogenic and thus contributes to obesity whereas metformin is associated with weight loss, making it difficult to interpret whether GLP-1 receptor agonist or the comparator medication is truly altering malignancy risk. Finally, insulin itself is associated with increased cancer risk due to affinity for the Insulin-like growth factor (IGF-1) receptor.^22^ It is for these reasons that we elected to compare GLP-1 receptor agonists to SGLT2 inhibitors, since both medications are used in diabetes of comparable severity, but differ significantly in their mechanism of action, with SGLT2 inhibitors not significantly affecting body adiposity.

Multiple myeloma develops from a preceding monoclonal gammopathy, a low-grade medullary plasma cell dyscrasia that has already acquired many of the genetic hallmarks of malignancy. The risk of progression to multiple myeloma is variable and associated with parameters reflecting disease severity, such as blood monoclonal protein level, bone marrow plasma cell frequency, and free light chain ratio. As risk stratification tools for MGUS and smoldering myeloma become more refined, early interventions, including with lenalidomide, daratumumab, or chimeric antigen receptor T (CAR-T) cell therapies, are increasingly investigated as means to slow or prevent the progression to overt malignancy.^23^^,^^24^ Obesity is one potential candidate that may mediate the transformation of MGUS into multiple myeloma. Previous large retrospective studies have noted 9–28% increased risk of multiple myeloma for every 5 kg/mˆ2 increase in BMI in early adulthood, particularly in those with persistently overweight or obese BMI values through adulthood.^25^, ^26^, ^27^ Obesity is thought to influence myelomagenesis through several mechanisms.^28^ The counter-regulatory hormone adiponectin, which is reduced both in obesity and those with MGUS who progress to myeloma, has anti-tumor activity and induces myeloma cell death.^29^^,^^30^ Bone marrow adipocytes, which have unique and poorly understood functions in hematopoiesis, are intimately associated with myeloma cells, and have been shown to promote their migration through chemokine release, provide fatty acids for use as metabolic substrates, prevent apoptosis via secretion of interleukin 6, interleukin-1b, and tumor necrosis factor alpha, and contribute to bone demineralization via suppression of osteoblast differentiation.^31^, ^32^, ^33^, ^34^, ^35^ Finally, a recent study found the enzyme Acetyl-COA Synthetase 2 (ACSS2), significantly overexpressed in multiple myeloma cells of obese patients, promotes plasma cello differentiation and survival by stabilization of the key transcription factor IRF4.^36^ Our data support a potential role for GLP-1 receptor agonists as a means to target these adipose-dependent pathways in the prevention of multiple myeloma, adding to previous studies reporting improved survival in myeloma patients taking metformin.^37^

For patients with diabetes mellitus and hematologic malignancies, the adverse effects of specific diabetes medications may present unacceptable risks, though there is a lack of research to guide decisions for such patients. Our results unexpectedly associate SGLT2 inhibitor use with a higher risk of mortality, an effect that was persistent in multiple myeloma and acute myeloid leukemia after controlling for both chronic kidney disease and congestive heart failure. The reason for this increased mortality is unclear but may be linked to two features of this medication class. Firstly, the increased risk for genitourinary infections may be more dangerous in patients with hematologic malignancies, who are immunosuppressed from their disease and treatment. Second, SGLT2 inhibitors, which tend to raise creatinine compared to other diabetes medications, may worsen kidney-associated complications in multiple myeloma. Indeed, a recent study of diabetics with multiple myeloma taking SGLT2 inhibitors, 42.4% of patients discontinued SGLT2 inhibitor use over a period of 15 months, with 21.4% of discontinuations being due to an increase in serum creatinine.^14^ These findings need to be carefully contextualized with other studies on the relationship between SGLT2 inhibitors and hematologic cancers, where it has been hypothesized that SGLT2 is a mechanism by which adult T cell leukemia cells obtain glucose for metabolic needs, with SGLT inhibitors representing a potential novel therapeutic option.^38^ However, to the best of our knowledge, there have been no large retrospective or prospective human studies on SGLT2 inhibitors and hematologic malignancies to date.

In conclusion, our review of a large multi-institutional US database of electronic health record data demonstrated that GLP-1 receptor agonist use was associated with a significant decrease in risk for multiple myeloma, but not acute myeloid leukemia, chronic myeloid leukemia, or myelodysplastic syndrome in patients with type 2 diabetes mellitus. In contrast, use of SGLT2 inhibitors in the same population did not modify risk of the studied hematologic malignancies but did increase the risk of death in patients with both type 2 diabetes mellitus and either acute myeloid leukemia or multiple myeloma, independently of heart failure.

## Contributors

EI: conceptualization, literature search, study design, data interpretation, writing-original draft, writing-review & editing, figures, project administration.

KP: accessed and verified data, data curation, data analysis, methodology, validation, figures.

JP: accessed and verified data, data curation, data analysis, methodology, validation.

KVB: conceptualization, study design, data interpretation, writing-review & editing.

## Data sharing statement

Requests for access to data related to this project should be directed to Dr. Koen van Besien at koen.vanbesien@uhhospitals.org.

## Declaration of interests

EI is funded by the American Society of Hematology to perform unrelated research work. EI received abstract support from the American Society of Hematology to present this work at the ASH Annual Meeting in 2025.

KVB is shareholder in Hemogenyx and Avertix. KVB is member of DSMB for Realta, Autolus, ADCT, research support Johnson & Johnson.

The remaining authors declare no conflicts of interest.
