# Supplementary material for: Incidence of hematologic malignancies and mortality associated with GLP-1 receptor agonist and SGLT2 inhibitor use in type 2 diabetes mellitus: results of a retrospective cohort study of electronic health records
Source: eClinicalMedicine. 2026 Jan 14;91:103749. doi: 10.1016/j.eclinm.2025.103749 (PMC12830215; doi:10.1016/j.eclinm.2025.103749)
Supplement: Supplementary Tables [file mmc1.docx]

|  | **Medication** | **Number of patients** | **Event n** | **HR^1^** | **95% CI^1^** | **p-value** |
| --- | --- | --- | --- | --- | --- | --- |
| **Acute myeloid leukemia** | **No Drug ^2^** | 51,786 | 133^3^ | -- | -- | -- |
|  | **GLP-1 RA** | 12,480 | 3 | 0.39 | 0.13, 1.20 | 0.102 |
|  | **No Drug ^2^** | 51,786 | 127 | -- | -- | -- |
|  | **SGLT2 inhibitor** | 10,680 | 8 | 1.22 | 0.57, 2.62 | 0.613 |
| **Multiple myeloma** | **No Drug ^2^** | 51,786 | 230^3^ | -- | -- | -- |
|  | **GLP-1 RA** | 12,480 | 4 | 0.33 | 0.12, 0.90 | **0.03** |
|  | **No Drug ^2^** | 51,786 | 229 | -- | -- | -- |
|  | **SGLT2 inhibitor** | 10,680 | 17 | 1.04 | 0.63, 1.72 | 0.885 |
| **Chronic myeloid leukemia** | **No Drug ^2^** | 51,786 | 46 | -- | -- | -- |
|  | **GLP-1 RA** | 12,480 | 2 | 0.88 | 0.21, 3.70 | 0.866 |
|  | **No Drug ^2^** | 51,786 | 50^3^ | -- | -- | -- |
|  | **SGLT2 inhibitor** | 10,680 | 5 | 1.51 | 0.58, 3.89 | 0.398 |
| **Myelodysplastic syndrome** | **No Drug ^2^** | 51,786 | 100 | -- | -- | -- |
|  | **GLP-1 RA** | 12,480 | 5 | 0.94 | 0.38, 2.32 | 0.892 |
|  | **No Drug ^2^** | 51,786 | 109^3^ | -- | -- | -- |
|  | **SGLT2 inhibitor** | 10,680 | 13 | 1.43 | 0.80, 2.53 | 0.225 |
| 1 HR = Hazard Ratio, CI = Confidence Interval; 2 Patients taking neither GLP-1 RA nor SGLT2 inhibitors; 3 Patients with malignancy diagnosis prior to medication were counted within the control events | | | | | | |

Supplemental table 1: Risk of hematologic malignancy in type 2 diabetes with HbA1c > 8% with GLP-1 RA or SGLT2 inhibitor use. Hazard ratio, confidence interval and p-values from a time-dependent Cox proportional hazards model for the outcomes of hematologic neoplasms in subjects with type 2 diabetes mellitus and HbA1c > 8% using GLP-1 RAs or SGLT2 inhibitors. Models were adjusted for race and ethnicity and weighted by age, sex, nicotine dependance, history of cerebral infarction, history of chronic ischemic heart disease, chronic kidney disease (CKD) and hypertension.

|  | **Medication** | **Number of patients** | **Event n** | **HR^1^** | **95% CI^1^** | **p-value** |
| --- | --- | --- | --- | --- | --- | --- |
| **Acute myeloid leukemia** | **No Drug ^2^** | 161,256 | 447^3^ | -- | -- | -- |
|  | **GLP-1 RA** | 41,208 | 14 | 0.77 | 0.45, 1.31 | 0.332 |
|  | **No Drug ^2^** | 161,256 | 445 | -- | -- | -- |
|  | **SGLT2 inhibitor** | 21,628 | 13 | 0.84 | 0.47, 1.50 | 0.557 |
| **Multiple myeloma** | **No Drug ^2^** | 161,256 | 896 | -- | -- | -- |
|  | **GLP-1 RA** | 41,208 | 24 | 0.64 | 0.42, 0.96 | **0.032** |
|  | **No Drug ^2^** | 161,256 | 910^3^ | -- | -- | -- |
|  | **SGLT2 inhibitor** | 21,628 | 44 | 1.07 | 0.77, 1.47 | 0.695 |
| **Chronic myeloid leukemia** | **No Drug ^2^** | 161,256 | 190 | -- | -- | -- |
|  | **GLP-1 RA** | 41,208 | 11 | 1.19 | 0.63, 2.23 | 0.598 |
|  | **No Drug ^2^** | 161,256 | 196^3^ | -- | -- | -- |
|  | **SGLT2 inhibitor** | 21,628 | 10 | 1.06 | 0.55, 2.05 | 0.857 |
| **Myelodysplastic syndrome** | **No Drug ^2^** | 161,256 | 521 | -- | -- | -- |
|  | **GLP-1 RA** | 41,208 | 26 | 1.16 | 0.77, 1.74 | 0.468 |
|  | **No Drug ^2^** | 161,256 | 528^3^ | -- | -- | -- |
|  | **SGLT2 inhibitor** | 21,628 | 26 | 1.13 | 0.75, 1.71 | 0.561 |
| 1 HR = Hazard Ratio, CI = Confidence Interval; 2 Patients taking neither GLP-1 RA nor SGLT2 inhibitors; 3 Patients with malignancy diagnosis prior to medication were counted within the control events | | | | | | |

Supplemental table 2: Risk of hematologic malignancy in type 2 diabetes with BMI > 30 with GLP-1 RA or SGLT2 inhibitor use. Hazard ratio, confidence interval and p-values from a time-dependent Cox proportional hazards model for the outcomes of hematologic neoplasms in subjects with type 2 diabetes mellitus and BMI > 30 using GLP-1 receptor agonists or SGLT2 inhibitors. Models were adjusted for race and ethnicity and weighted by age, sex, nicotine dependance, history of cerebral infarction, history of chronic ischemic heart disease, chronic kidney disease (CKD) and hypertension.

| **Malignancy** |  | **HR^1^** | **95% CI^1^** | **p-value** |
| --- | --- | --- | --- | --- |
| **Multiple myeloma** | **GLP-1 RA** | 0.66 | 0.47, 0.93 | **0.018** |
| **Multiple myeloma (HbA1c >8)** | **GLP-1 RA** | 0.33 | 0.12, 0.89 | **0.028** |
| **Multiple myeloma (BMI >30)** | **GLP-1 RA** | 0.67 | 0.45, 1.01 | 0.056 |
| 1 HR = Hazard Ratio, CI = Confidence Interval; Reference level for Medication (No medication); covariates included but not shown Race (ref = White), Ethnicity (ref=Not Hispanic or Latino), age, sex (ref = Male), nicotine dependance (ref = No), history of cerebral infarction (ref = No), history of chronic ischemic heart disease (ref = No), chronic kidney disease (CKD; ref = No) and hypertension (ref = No); Patients were weighted by age, sex, nicotine dependence, history of cerebral infarction, history of chronic ischemic heart disease, chronic kidney disease (CKD) and hypertension | | | | |

Supplemental table 3. Risk of multiple myeloma in type 2 diabetes with GLP-1 RA use after adjustment for covariates. Hazard ratio, confidence interval and p-values from a time-dependent Cox proportional hazards model for the outcome of multiple myeloma in subjects with type 2 diabetes mellitus using GLP-1 agonists. Results are presented from main analysis and subgroup analysis for patients with HbA1c > 8% and patients with BMI > 30. Models were adjusted for race, ethnicity, age, sex, nicotine dependance, history of cerebral infarction, history of chronic ischemic heart disease, chronic kidney disease (CKD) and hypertension and weighted by age, sex, nicotine dependance, history of cerebral infarction, history of chronic ischemic heart disease, chronic kidney disease (CKD) and hypertension. Significant p-values in bold.

| **Characteristic** | **Overall**  **N = 875^1^** | **No Medication Use**  **N = 854^1^** | **GLP-1 receptor agonist Use**  **N = 21^1^** | **p-value^2^** |
| --- | --- | --- | --- | --- |
| **Age** | 59.4 ± 15.1 | 59.5 ± 15.1 | 58.3 ± 14.8 | 0.719 |
| **Sex** |  |  |  | 0.228 |
| Male | 488 (55.8%) | 479 (56.1%) | 9 (42.9%) |  |
| Female | 387 (44.2%) | 375 (43.9%) | 12 (57.1%) |  |
| **Race** |  |  |  | 0.61 |
| White | 653 (74.6%) | 637 (74.6%) | 16 (76.2%) |  |
| Black or African American | 120 (13.7%) | 116 (13.6%) | 4 (19.0%) |  |
| Other Race | 102 (11.7%) | 101 (11.8%) | 1 (4.8%) |  |
| **Ethnicity** |  |  |  | >0.999 |
| Not Hispanic or Latino | 793 (90.6%) | 774 (90.6%) | 19 (90.5%) |  |
| Hispanic or Latino | 82 (9.4%) | 80 (9.4%) | 2 (9.5%) |  |
| **Nicotine dependance** | 327 (37.4%) | 323 (37.8%) | 4 (19.0%) | 0.079 |
| **BMI** | 30.3 ± 7.3 | 30.2 ± 7.3 | 34.7 ± 6.0 | **0.003** |
| **A1c** | 6.7 ± 1.8 | 6.7 ± 1.8 | 7.2 ± 1.5 | 0.15 |
| **Cerebral infarction** | 69 (7.9%) | 67 (7.8%) | 2 (9.5%) | 0.678 |
| **Chronic ischemic heart disease** | 174 (19.9%) | 169 (19.8%) | 5 (23.8%) | 0.587 |
| **Chronic kidney disease (CKD)** | 141 (16.1%) | 137 (16.0%) | 4 (19.0%) | 0.762 |
| **Essential (primary) hypertension** | 461 (52.7%) | 451 (52.8%) | 10 (47.6%) | 0.638 |
| **Heart failure** | 107 (12.2%) | 104 (12.2%) | 3 (14.3%) | 0.734 |
| 1 n (%); Mean ± SD; 2 Pearson’s Chi-squared test; Welch Two Sample t-test | | | | |

Supplemental table 4: Baseline characteristics of subjects with diabetes mellitus and acute myeloid leukemia who use GLP-1 receptor agonists or neither GLP-1 receptor agonists or SGLT2 inhibitors. P-values are shown comparing users of a particular medication class to those who do not use either.

| **Characteristic** | **Overall**  **N = 888^1^** | **No Medication Use**  **N = 854^1^** | **SGLT2 Inhibitor Use**  **N = 34^1^** | **p-value^2^** |
| --- | --- | --- | --- | --- |
| **Age** | 59.7 ± 15.0 | 59.5 ± 15.1 | 65.0 ± 9.5 | **0.003** |
| **Sex** |  |  |  | 0.513 |
| Male | 500 (56.3%) | 479 (56.1%) | 21 (61.8%) |  |
| Female | 388 (43.7%) | 375 (43.9%) | 13 (38.2%) |  |
| **Race** |  |  |  | 0.189 |
| White | 661 (74.4%) | 637 (74.6%) | 24 (70.6%) |  |
| Black or African American | 124 (14.0%) | 116 (13.6%) | 8 (23.5%) |  |
| Other Race | 103 (11.6%) | 101 (11.8%) | 2 (5.9%) |  |
| **Ethnicity** |  |  |  | 0.356 |
| Not Hispanic or Latino | 807 (90.9%) | 774 (90.6%) | 33 (97.1%) |  |
| Hispanic or Latino | 81 (9.1%) | 80 (9.4%) | 1 (2.9%) |  |
| **Nicotine dependance** | 335 (37.7%) | 323 (37.8%) | 12 (35.3%) | 0.766 |
| **BMI** | 30.3 ± 7.3 | 30.2 ± 7.3 | 31.2 ± 6.9 | 0.441 |
| **A1c** | 6.7 ± 1.8 | 6.7 ± 1.8 | 7.4 ± 2.0 | 0.054 |
| **Cerebral infarction** | 70 (7.9%) | 67 (7.8%) | 3 (8.8%) | 0.745 |
| **Chronic ischemic heart disease** | 177 (19.9%) | 169 (19.8%) | 8 (23.5%) | 0.592 |
| **Chronic kidney disease (CKD)** | 145 (16.3%) | 137 (16.0%) | 8 (23.5%) | 0.247 |
| **Essential (primary) hypertension** | 469 (52.8%) | 451 (52.8%) | 18 (52.9%) | 0.988 |
| **Heart failure** | 113 (12.7%) | 104 (12.2%) | 9 (26.5%) | **0.03** |
| 1 n (%); Mean ± SD; 2 Pearson’s Chi-squared test; Welch Two Sample t-test | | | | |

Supplemental table 5: Baseline characteristics of subjects with diabetes mellitus and acute myeloid leukemia who use SGLT2 inhibitors or neither GLP-1 receptor agonists or SGLT2 inhibitors. P-values are shown comparing users of a particular medication class to those who do not use either.

| **Characteristic** | **Overall**  **N = 1,750^1^** | **No Medication Use**  **N = 1,716^1^** | **GLP-1 receptor agonist Use**  **N = 34^1^** | **p-value^2^** |
| --- | --- | --- | --- | --- |
| **Age** | 65.4 ± 11.2 | 65.4 ± 11.2 | 61.6 ± 11.7 | 0.068 |
| **Sex** |  |  |  | **0.015** |
| Male | 926 (52.9%) | 915 (53.3%) | 11 (32.4%) |  |
| Female | 824 (47.1%) | 801 (46.7%) | 23 (67.6%) |  |
| **Race** |  |  |  | 0.366 |
| White | 1,151 (65.8%) | 1,125 (65.6%) | 26 (76.5%) |  |
| Black or African American | 437 (25.0%) | 432 (25.2%) | 5 (14.7%) |  |
| Other Race | 162 (9.3%) | 159 (9.3%) | 3 (8.8%) |  |
| **Ethnicity** |  |  |  | 0.106 |
| Not Hispanic or Latino | 1,617 (92.4%) | 1,583 (92.2%) | 34 (100.0%) |  |
| Hispanic or Latino | 133 (7.6%) | 133 (7.8%) | 0 (0.0%) |  |
| **Nicotine dependance** | 700 (40.0%) | 689 (40.2%) | 11 (32.4%) | 0.358 |
| **BMI** | 30.2 ± 6.8 | 30.2 ± 6.8 | 34.0 ± 6.0 | **<0.001** |
| **A1c** | 6.5 ± 1.7 | 6.5 ± 1.7 | 6.8 ± 1.4 | 0.227 |
| **Cerebral infarction** | 157 (9.0%) | 155 (9.0%) | 2 (5.9%) | 0.763 |
| **Chronic ischemic heart disease** | 372 (21.3%) | 367 (21.4%) | 5 (14.7%) | 0.346 |
| **Chronic kidney disease (CKD)** | 413 (23.6%) | 408 (23.8%) | 5 (14.7%) | 0.217 |
| **Essential (primary) hypertension** | 993 (56.7%) | 976 (56.9%) | 17 (50.0%) | 0.423 |
| **Heart failure** | 236 (13.5%) | 232 (13.5%) | 4 (11.8%) | >0.999 |
| 1 n (%); Mean ± SD; 2 Pearson’s Chi-squared test; Welch Two Sample t-test | | | | |

Supplemental table 6: Baseline characteristics of subjects with diabetes mellitus and multiple myeloma who use GLP-1 receptor agonists or neither GLP-1 receptor agonists or SGLT2 inhibitors. P-values are shown comparing users of a particular medication class to those who do not use either.

| **Characteristic** | **Overall**  **N = 1,799^1^** | **No Medication Use**  **N = 1,716^1^** | **SGLT2 Inhibitor Use**  **N = 83^1^** | **p-value^2^** |
| --- | --- | --- | --- | --- |
| **Age** | 65.4 ± 11.1 | 65.4 ± 11.2 | 65.5 ± 10.5 | 0.949 |
| **Sex** |  |  |  | **0.036** |
| Male | 969 (53.9%) | 915 (53.3%) | 54 (65.1%) |  |
| Female | 830 (46.1%) | 801 (46.7%) | 29 (34.9%) |  |
| **Race** |  |  |  | 0.334 |
| White | 1,177 (65.4%) | 1,125 (65.6%) | 52 (62.7%) |  |
| Black or African American | 458 (25.5%) | 432 (25.2%) | 26 (31.3%) |  |
| Other Race | 164 (9.1%) | 159 (9.3%) | 5 (6.0%) |  |
| **Ethnicity** |  |  |  | 0.325 |
| Not Hispanic or Latino | 1,662 (92.4%) | 1,583 (92.2%) | 79 (95.2%) |  |
| Hispanic or Latino | 137 (7.6%) | 133 (7.8%) | 4 (4.8%) |  |
| **Nicotine dependance** | 715 (39.7%) | 689 (40.2%) | 26 (31.3%) | 0.109 |
| **BMI** | 30.2 ± 6.7 | 30.2 ± 6.8 | 31.2 ± 5.7 | 0.103 |
| **A1c** | 6.6 ± 1.7 | 6.5 ± 1.7 | 7.3 ± 2.1 | **<0.001** |
| **Cerebral infarction** | 159 (8.8%) | 155 (9.0%) | 4 (4.8%) | 0.187 |
| **Chronic ischemic heart disease** | 388 (21.6%) | 367 (21.4%) | 21 (25.3%) | 0.397 |
| **Chronic kidney disease (CKD)** | 420 (23.3%) | 408 (23.8%) | 12 (14.5%) | **0.05** |
| **Essential (primary) hypertension** | 1,016 (56.5%) | 976 (56.9%) | 40 (48.2%) | 0.119 |
| **Heart failure** | 245 (13.6%) | 232 (13.5%) | 13 (15.7%) | 0.578 |
| 1 n (%); Mean ± SD; 2 Pearson’s Chi-squared test; Welch Two Sample t-test | | | | |

Supplemental table 7: Baseline characteristics of subjects with diabetes mellitus and multiple myeloma who use SGLT2 inhibitors or neither GLP-1 receptor agonists or SGLT2 inhibitors. P-values are shown comparing users of a particular medication class to those who do not use either.

| **Characteristic** | **Overall**  **N = 320^1^** | **No Medication Use**  **N = 307^1^** | **GLP-1 receptor agonist Use**  **N = 13^1^** | **p-value^2^** |
| --- | --- | --- | --- | --- |
| **Age** | 61.4 ± 14.1 | 61.7 ± 14.1 | 53.5 ± 13.3 | **0.049** |
| **Sex** |  |  |  | 0.804 |
| Male | 183 (57.2%) | 176 (57.3%) | 7 (53.8%) |  |
| Female | 137 (42.8%) | 131 (42.7%) | 6 (46.2%) |  |
| **Race** |  |  |  | 0.176 |
| White | 237 (74.1%) | 228 (74.3%) | 9 (69.2%) |  |
| Black or African American | 50 (15.6%) | 46 (15.0%) | 4 (30.8%) |  |
| Other Race | 33 (10.3%) | 33 (10.7%) | 0 (0.0%) |  |
| **Ethnicity** |  |  |  | >0.999 |
| Not Hispanic or Latino | 300 (93.8%) | 287 (93.5%) | 13 (100.0%) |  |
| Hispanic or Latino | 20 (6.2%) | 20 (6.5%) | 0 (0.0%) |  |
| **Nicotine dependance** | 101 (31.6%) | 98 (31.9%) | 3 (23.1%) | 0.762 |
| **BMI** | 31.3 ± 7.0 | 31.1 ± 6.9 | 36.7 ± 5.8 | **0.004** |
| **A1c** | 6.8 ± 1.8 | 6.8 ± 1.8 | 7.0 ± 1.0 | 0.609 |
| **Cerebral infarction** | 21 (6.6%) | 21 (6.8%) | 0 (0.0%) | >0.999 |
| **Chronic ischemic heart disease** | 77 (24.1%) | 75 (24.4%) | 2 (15.4%) | 0.741 |
| **Chronic kidney disease (CKD)** | 52 (16.2%) | 51 (16.6%) | 1 (7.7%) | 0.701 |
| **Essential (primary) hypertension** | 175 (54.7%) | 168 (54.7%) | 7 (53.8%) | 0.95 |
| **Heart failure** | 30 (9.4%) | 29 (9.4%) | 1 (7.7%) | >0.999 |
| 1 n (%); Mean ± SD; 2 Pearson’s Chi-squared test; Welch Two Sample t-test | | | | |

Supplemental table 8: Baseline characteristics of subjects with diabetes mellitus and chronic myeloid leukemia who use GLP-1 receptor agonists or neither GLP-1 receptor agonists or SGLT2 inhibitors. P-values are shown comparing users of a particular medication class to those who do not use either.

| **Characteristic** | **Overall**  **N = 323^1^** | **No Medication Use**  **N = 307^1^** | **SGLT2 Inhibitor Use**  **N = 16^1^** | **p-value^2^** |
| --- | --- | --- | --- | --- |
| **Age** | 61.5 ± 13.9 | 61.7 ± 14.1 | 58.5 ± 10.8 | 0.269 |
| **Sex** |  |  |  | 0.162 |
| Male | 188 (58.2%) | 176 (57.3%) | 12 (75.0%) |  |
| Female | 135 (41.8%) | 131 (42.7%) | 4 (25.0%) |  |
| **Race** |  |  |  | 0.686 |
| White | 241 (74.6%) | 228 (74.3%) | 13 (81.2%) |  |
| Black or African American | 47 (14.6%) | 46 (15.0%) | 1 (6.2%) |  |
| Other Race | 35 (10.8%) | 33 (10.7%) | 2 (12.5%) |  |
| **Ethnicity** |  |  |  | 0.096 |
| Not Hispanic or Latino | 300 (92.9%) | 287 (93.5%) | 13 (81.2%) |  |
| Hispanic or Latino | 23 (7.1%) | 20 (6.5%) | 3 (18.8%) |  |
| **Nicotine dependance** | 103 (31.9%) | 98 (31.9%) | 5 (31.2%) | 0.955 |
| **BMI** | 31.0 ± 6.9 | 31.1 ± 6.9 | 29.8 ± 5.9 | 0.413 |
| **A1c** | 6.9 ± 1.8 | 6.8 ± 1.8 | 8.0 ± 2.0 | 0.028 |
| **Cerebral infarction** | 22 (6.8%) | 21 (6.8%) | 1 (6.2%) | >0.999 |
| **Chronic ischemic heart disease** | 77 (23.8%) | 75 (24.4%) | 2 (12.5%) | 0.375 |
| **Chronic kidney disease (CKD)** | 52 (16.1%) | 51 (16.6%) | 1 (6.2%) | 0.484 |
| **Essential (primary) hypertension** | 171 (52.9%) | 168 (54.7%) | 3 (18.8%) | **0.005** |
| **Heart failure** | 32 (9.9%) | 29 (9.4%) | 3 (18.8%) | 0.203 |
| 1 n (%); Mean ± SD; 2 Pearson’s Chi-squared test; Welch Two Sample t-test | | | | |

Supplemental table 9: Baseline characteristics of subjects with diabetes mellitus and chronic myeloid leukemia who use SGLT2 inhibitors or neither GLP-1 receptor agonists or SGLT2 inhibitors. P-values are shown comparing users of a particular medication class to those who do not use either.

| **Characteristic** | **Overall**  **N = 1,179^1^** | **No Medication Use**  **N = 1,145^1^** | **GLP-1 receptor agonist Use**  **N = 34^1^** | **p-value^2^** |
| --- | --- | --- | --- | --- |
| **Age** | 66.0 ± 13.9 | 66.2 ± 13.9 | 59.7 ± 13.5 | **0.009** |
| **Sex** |  |  |  | **0.029** |
| Male | 667 (56.6%) | 654 (57.1%) | 13 (38.2%) |  |
| Female | 512 (43.4%) | 491 (42.9%) | 21 (61.8%) |  |
| **Race** |  |  |  | 0.126 |
| White | 943 (80.0%) | 918 (80.2%) | 25 (73.5%) |  |
| Black or African American | 148 (12.6%) | 140 (12.2%) | 8 (23.5%) |  |
| Other Race | 88 (7.5%) | 87 (7.6%) | 1 (2.9%) |  |
| **Ethnicity** |  |  |  | >0.999 |
| Not Hispanic or Latino | 1,093 (92.7%) | 1,061 (92.7%) | 32 (94.1%) |  |
| Hispanic or Latino | 86 (7.3%) | 84 (7.3%) | 2 (5.9%) |  |
| **Nicotine dependance** | 482 (40.9%) | 470 (41.0%) | 12 (35.3%) | 0.501 |
| **BMI** | 29.9 ± 6.9 | 29.7 ± 6.8 | 37.0 ± 9.2 | **<0.001** |
| **A1c** | 6.3 ± 1.5 | 6.3 ± 1.5 | 6.7 ± 1.5 | 0.085 |
| **Cerebral infarction** | 124 (10.5%) | 120 (10.5%) | 4 (11.8%) | 0.775 |
| **Chronic ischemic heart disease** | 327 (27.7%) | 322 (28.1%) | 5 (14.7%) | 0.085 |
| **Chronic kidney disease (CKD)** | 268 (22.7%) | 261 (22.8%) | 7 (20.6%) | 0.762 |
| **Essential (primary) hypertension** | 669 (56.7%) | 652 (56.9%) | 17 (50.0%) | 0.421 |
| **Heart failure** | 194 (16.5%) | 188 (16.4%) | 6 (17.6%) | 0.849 |
| 1 n (%); Mean ± SD; 2 Pearson’s Chi-squared test; Welch Two Sample t-test | | | | |

Supplemental table 10: Baseline characteristics of subjects with diabetes mellitus and myelodysplastic syndrome who use GLP-1 receptor agonists or neither GLP-1 receptor agonists or SGLT2 inhibitors. P-values are shown comparing users of a particular medication class to those who do not use either.

| **Characteristic** | **Overall**  **N = 1,192^1^** | **No Medication Use**  **N = 1,145^1^** | **SGLT2 Inhibitor Use**  **N = 47^1^** | **p-value^2^** |
| --- | --- | --- | --- | --- |
| **Age** | 66.3 ± 13.8 | 66.2 ± 13.9 | 68.4 ± 12.0 | 0.237 |
| **Sex** |  |  |  | 0.362 |
| Male | 684 (57.4%) | 654 (57.1%) | 30 (63.8%) |  |
| Female | 508 (42.6%) | 491 (42.9%) | 17 (36.2%) |  |
| **Race** |  |  |  | 0.961 |
| White | 956 (80.2%) | 918 (80.2%) | 38 (80.9%) |  |
| Black or African American | 146 (12.2%) | 140 (12.2%) | 6 (12.8%) |  |
| Other Race | 90 (7.6%) | 87 (7.6%) | 3 (6.4%) |  |
| **Ethnicity** |  |  |  | 0.773 |
| Not Hispanic or Latino | 1,104 (92.6%) | 1,061 (92.7%) | 43 (91.5%) |  |
| Hispanic or Latino | 88 (7.4%) | 84 (7.3%) | 4 (8.5%) |  |
| **Nicotine dependance** | 485 (40.7%) | 470 (41.0%) | 15 (31.9%) | 0.212 |
| **BMI** | 29.8 ± 6.8 | 29.7 ± 6.8 | 31.6 ± 6.4 | 0.052 |
| **A1c** | 6.3 ± 1.5 | 6.3 ± 1.5 | 7.6 ± 1.7 | **<0.001** |
| **Cerebral infarction** | 122 (10.2%) | 120 (10.5%) | 2 (4.3%) | 0.221 |
| **Chronic ischemic heart disease** | 331 (27.8%) | 322 (28.1%) | 9 (19.1%) | 0.178 |
| **Chronic kidney disease (CKD)** | 267 (22.4%) | 261 (22.8%) | 6 (12.8%) | 0.106 |
| **Essential (primary) hypertension** | 671 (56.3%) | 652 (56.9%) | 19 (40.4%) | **0.025** |
| **Heart failure** | 198 (16.6%) | 188 (16.4%) | 10 (21.3%) | 0.381 |
| 1 n (%); Mean ± SD; 2 Pearson’s Chi-squared test; Welch Two Sample t-test | | | | |

Supplemental table 11: Baseline characteristics of subjects with diabetes mellitus and myelodysplastic syndrome who use SGLT2 inhibitors or neither GLP-1 receptor agonists or SGLT2 inhibitors. P-values are shown comparing users of a particular medication class to those who do not use either.

|  |  | **Heart failure as Covariate** | | | **Without heart failure** | | | **With heart failure** | | |
| --- | --- | --- | --- | --- | --- | --- | --- | --- | --- | --- |
|  |  |  |  |  |  |  |  |  |  |  |
|  | **Event rate, n (%)** | **HR1** | **95% CI1** | **p-value** | **HR1** | **95% CI1** | **p-value** | **HR1** | **95% CI1** | **p-value** |
|  |  |  |  |  |  |  |  |  |  |  |
| **SGLT2 Inhibitor *** |  |  |  |  |  |  |  |  |  |  |
| No medication |  | — | — | — | — | — | — | — | — | — |
| Medication | 22 (5.26%) | 2.27 | 1.41, 3.65 | **<0.001** | 2.14 | 1.25, 3.66 | **0.006** | 2.62 | 1.10, 6.24 | **0.03** |
| **Heart failure** |  |  |  |  |  |  |  |  |  |  |
| Yes |  | 1.88 | 1.11, 3.17 | **0.019** | — | — | — | — | — | — |
|  | | | | | | | | | | |

Supplemental table 12: Risk of mortality associated with SGLT2 inhibitor use in diabetics with multiple myeloma, with or without heart failure. Results from a Cox proportional hazards model with the outcome of mortality or last follow-up in patients with diabetes mellitus and multiple myeloma who do or do not take SGLT2 inhibitors. Heart failure is shown as a covariate, and separate analyses are shown for individuals with or without a diagnosis of heart failure.

| **Malignancy** |  | **HR^1^** | **95% CI^1^** | **p-value** |
| --- | --- | --- | --- | --- |
| **Acute myeloid leukemia** | **SGLT2i** | 2.47 | 1.46, 4.16 | **<0.001** |
| **Multiple myeloma** | **SGLT2i** | 2.44 | 1.51, 3.96 | **<0.001** |
| **Chronic myeloid leukemia** | **SGLT2i** | 3.46 | 1.15, 10.4 | **0.027** |
| 1 HR = Hazard Ratio, CI = Confidence Interval; * No patients with outcome and on medication; Reference level for Medication (No medication); covariates included but not shown Race (ref = White), Ethnicity (ref=Not Hispanic or Latino), heart failure (ref = No), age, sex (ref = Male), nicotine dependance (ref = No), history of cerebral infarction (ref = No), history of chronic ischemic heart disease (ref = No), chronic kidney disease (CKD, ref = No) and hypertension (ref = No); Patients were weighted by age, sex, nicotine dependance, history of cerebral infarction, history of chronic ischemic heart disease, chronic kidney disease (CKD) and hypertension; | | | | |

Supplemental table 13. Risk of mortality by use of SGLT2 inhibitors in type 2 diabetes with concurrent diagnosis of hematologic malignancy after adjustment for covariates. Hazard ratio, confidence interval and p-values from a time-dependent Cox proportional hazards model for the outcome of mortality in subjects with type 2 diabetes mellitus and the indicated malignancies using SGLT2 inhibitors. Models were adjusted for race, ethnicity, age, sex, nicotine dependance, history of cerebral infarction, history of chronic ischemic heart disease, chronic kidney disease (CKD) and hypertension and weighted by age, sex, nicotine dependance, history of cerebral infarction, history of chronic ischemic heart disease, chronic kidney disease (CKD) and hypertension. Table presents hazard ratio, 95% confidence interval and event rate as percent of total observations. Significant p-values in bold.


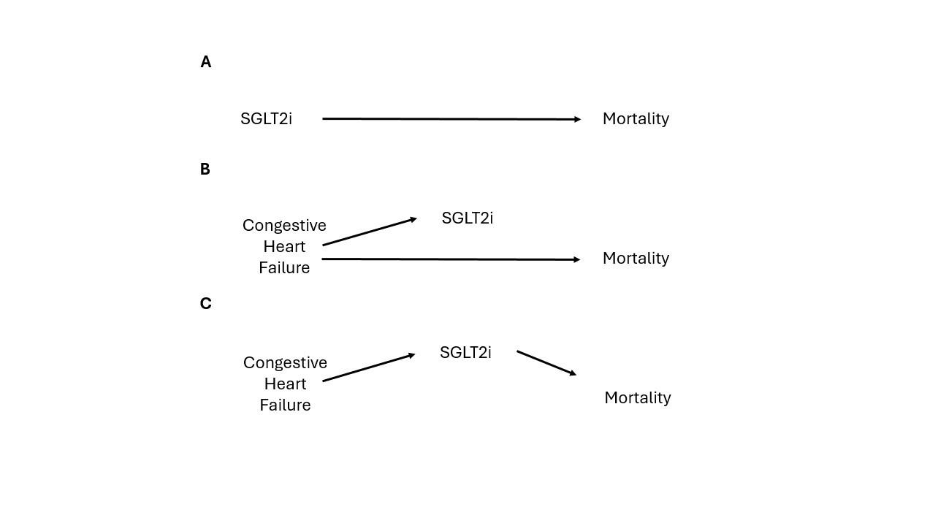


Supplemental Figure 1. Directed Acyclic Graph of Congestive Heart Failure, SGLT2 Inhibitor Use, and Mortality. A simple directed acyclic graph is shown for the hypothesized variables associated with mortality in individuals diagnosed with type 2 diabetes mellitus and hematologic malignancies of interest. (A) In AML, SGLT2 inhibitor use was independently associated with increased mortality but congestive heart failure was not. (B) In CML, congestive heart failure was associated with a significantly increased risk for mortality and individuals without congestive heart failure did not have elevated mortality risk if using SGLT2 inhibitors. (C) In MM, congestive heart failure was not associated with increased risk for mortality after adjustment for metabolic covariates, but individuals without congestive heart failure who used SGLT2 inhibitors still had an elevated risk of mortality.
